# Supplementary material for: Association between vmPFC gray matter volume and smoking initiation in adolescents
Source: Nat Commun. 2023 Aug 15;14:4684. doi: 10.1038/s41467-023-40079-2 (PMC10427673; doi:10.1038/s41467-023-40079-2)
Supplement: Supplementary file 1 — Supplementary Information [file 41467_2023_40079_MOESM1_ESM.pdf]

Supplementary Figures

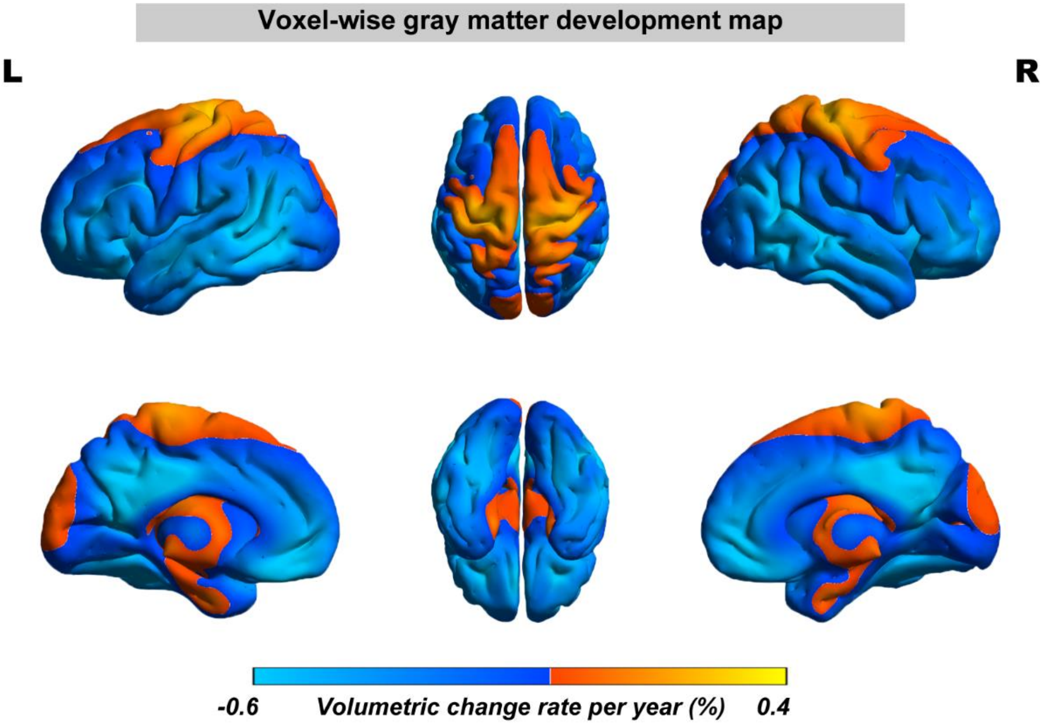

Fig. S1 The average gray matter volumetric change rate map. Red represented volume increasing and blue represented volume reduction. The brighter colour indicated the greater changes.

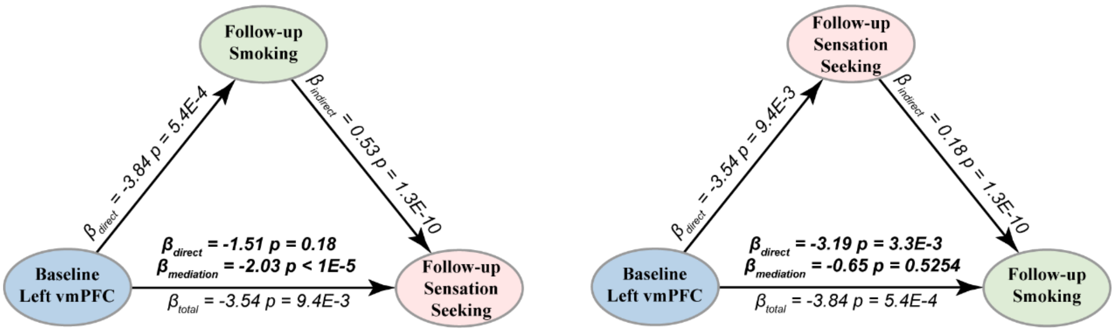

Fig. S2 The results of mediation analysis. The p-values of the mediation effects were obtained with 10000 times bootstrap samplings. The other two-tailed p-values were obtained with the linear regressions.

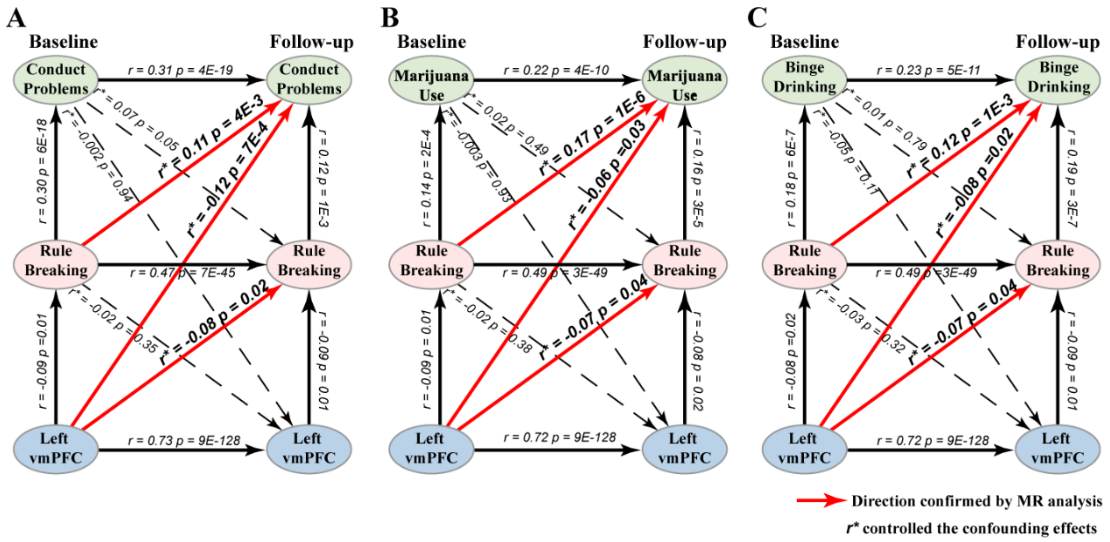

Fig. S3 The causal inference between the grey matter volume of left vmPFC and rule-breaking behaviour as well as addictive behaviour. a. Conduct problems; **b.** Marijuana use; **c.** Binge drinking. Solid lines represented significant associations, and red lines represented causal inference confirmed with MR. Also, see Table S6. The two-tailed p-values obtained in a longitudinal cross-lagged analyses were provided.

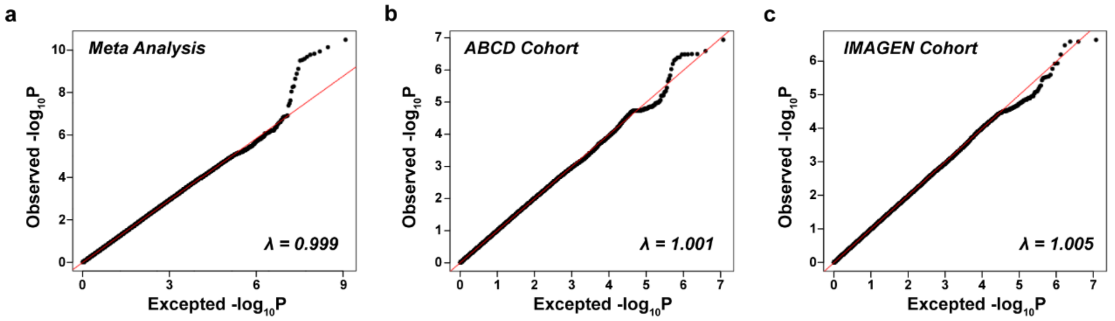

Fig. S4 The Q-Q plots for the GWAS of gray matter volume in the left vmPFC. Plotted results of **a.** the meta-analysis, **b.** the ABCD cohort, and **c.** the IMAGEN cohort.

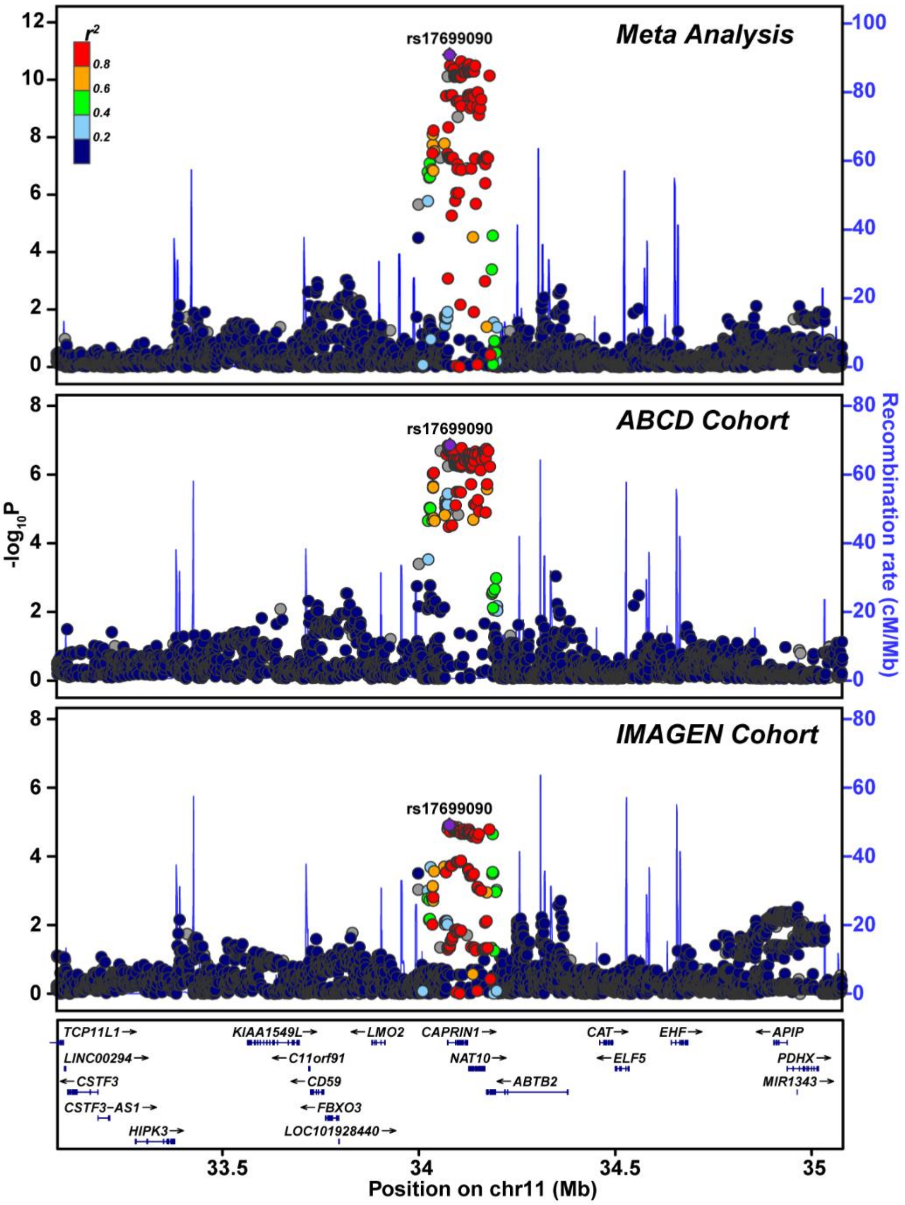

Fig. S5 The locus plot for the lead SNP (rs17699090, within *CAPRIN1*; associated with the gray matter volume of left vmPFC). Upper: the results of meta-analysis; middle: GWAS in the ABCD cohort; lower: GWAS in the IMAGEN cohort.

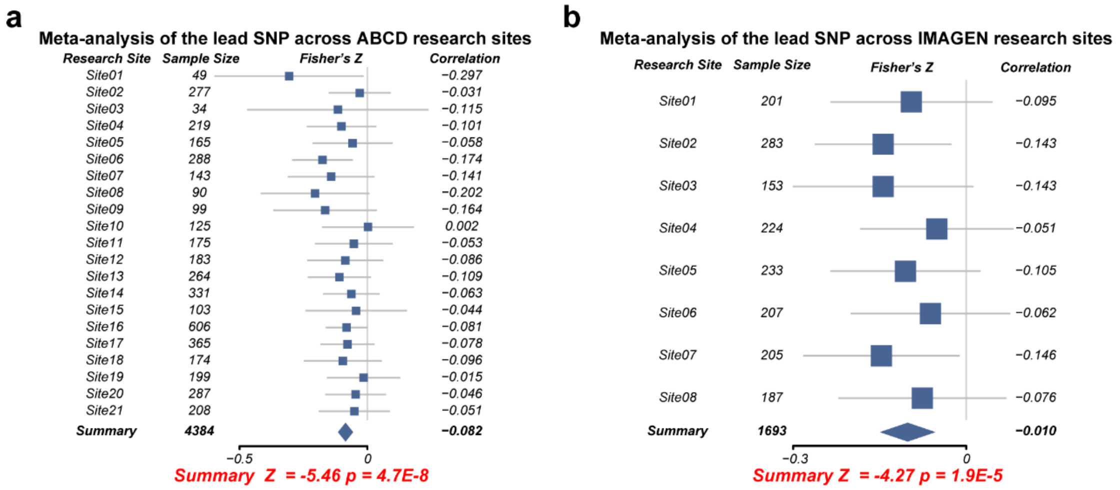

Fig. S6 The forest plots for meta-analyses of the lead SNP rs17699090's correlations with gray matter volume of the left vmPFC across research sites in both (a) ABCD and (b) IMAGEN cohorts. The squares represented the z-value of the correlation coefficient after the Fisher r to z transformation. The whiskers represented the 95% confidence intervals.

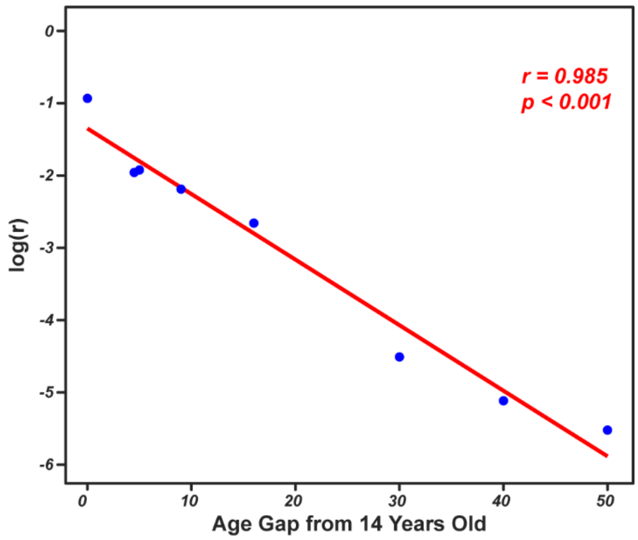

Fig. S7 The predictive effects of PRS (i.e., the correlation r) reduced exponentially (plot as log(r)) with increased age gaps.

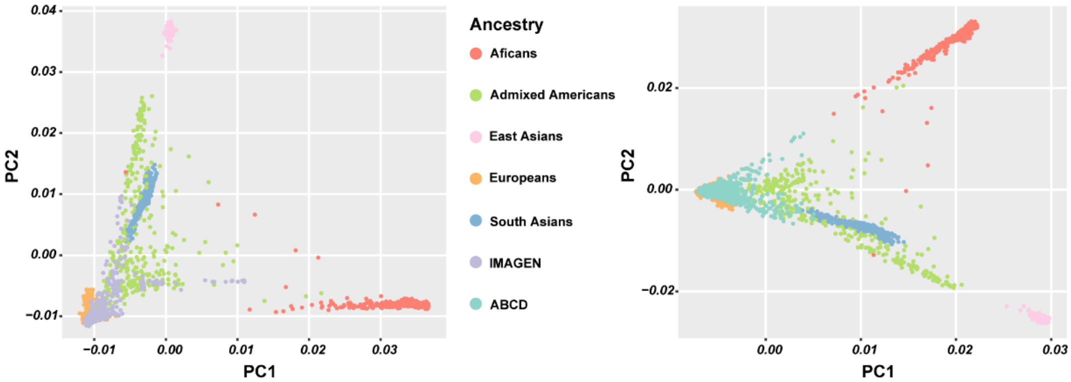

Fig. S8 Plots of first two components of IMAGEN (left) and ABCD (right) projected to 1000 Genome ancestry groups.

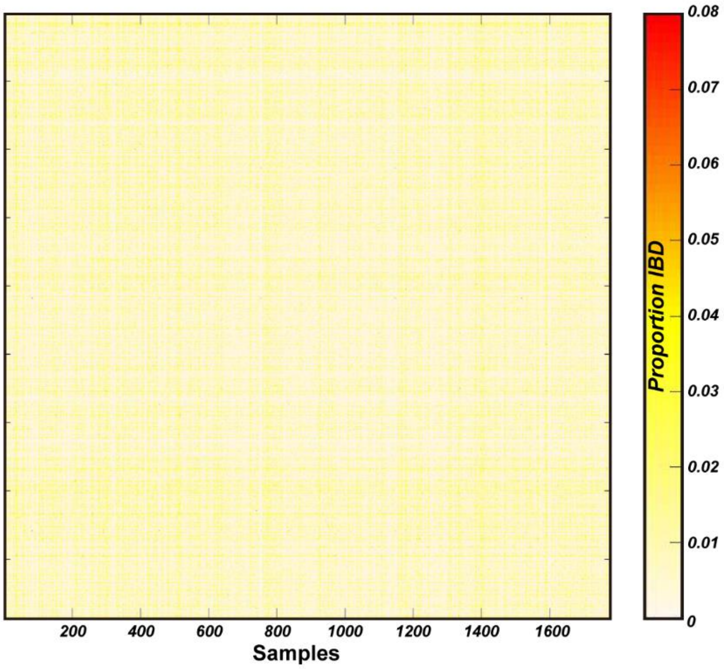

Fig. S9 The pairwise co-ancestry levels (i.e., the proportion of shared genome estimated using PLINK 2.0) between participants from the IMAGEN cohort.

Supplementary Tables

Table S1. Summary statistics of gray matter volume and development in clusters.

Summary Statistics

Clusters		Con Group (*n = 260*)		BL-S Group (*n = 181*)		FU-S Group (*n = 366*)	
		mean	*std*	*mean*	*std*	*mean*	*std*
Baseline	Left vmPFC	0.600	0.093	0.577	0.097	0.578	0.089
	Right vmPFC	0.485	0.083	0.473	0.086	0.477	0.086
Development	Left vmPFC	-4.12E-03	2.19E-03	-3.98E-03	1.97E-03	-4.20E-03	2.05E-03
	Right vmPFC	-2.79E-03	1.80E-03	-2.98E-03	1.87E-03	-3.27E-03	1.94E-03
Follow-up	Left vmPFC	0.581	0.080	0.540	0.093	0.555	0.079
	Right vmPFC	0.466	0.060	0.436	0.074	0.453	0.064

Group Comparison

Clusters		BL-S *vs* Con Group			FU-S *vs* Con Group		
		T	*p*	*Cohen's d*	*T*	*p*	*Cohen's d*
Baseline	Left vmPFC	**-2.539**	**0.011**	**-0.250**	**-3.837**	**1.4E-4**	**-0.315**
	Right vmPFC	**-1.968**	**0.049**	**-0.194**	-0.787	0.747	-0.065
Development	Left vmPFC	0.723	0.470	0.071	-0.996	0.319	-0.082
	Right vmPFC	**-2.039**	**0.042**	**-0.201**	**-4.426**	**1.1E-5**	**-0.363**
Follow-up	Left vmPFC	**-3.040**	**0.003**	**-0.299**	**-3.764**	**1.8E-4**	**-0.309**
	Right vmPFC	**-2.586**	**0.010**	**-0.255**	**-2.078**	**0.038**	**-0.171**

Among Males

Summary Statistics

Clusters		Con Group (*n = 106*)		BL-S Group (*n = 84*)		FU-S Group (*n = 173*)	
		mean	*std*	*mean*	*std*	*mean*	*std*
Baseline	Left vmPFC	0.599	0.095	0.578	0.098	0.573	0.091
	Right vmPFC	0.487	0.091	0.469	0.090	0.479	0.082
Development	Left vmPFC	-3.09E-03	1.85E-03	-3.08E-03	1.44E-03	-3.35E-03	1.73E-03
	Right vmPFC	-2.50E-03	1.64E-03	-2.69E-03	1.31E-03	-3.54E-03	1.79E-03
Follow-up	Left vmPFC	0.577	0.077	0.526	0.094	0.544	0.076
	Right vmPFC	0.465	0.061	0.426	0.078	0.447	0.065

Group Comparison

Clusters		BL-S *vs* Con Group			FU-S *vs* Con Group		
		T	$p_{one\text{-}tailed}$	*Cohen's d*	*T*	$p_{one\text{-}tailed}$	*Cohen's d*
Baseline	Left vmPFC	**-1.913**	**0.029**	**-0.279**	**-3.145**	**0.001**	**-0.388**
	Right vmPFC	**-1.956**	**0.026**	**-0.286**	-1.496	0.068	-0.185
Development	Left vmPFC	0.668	0.253	0.098	-0.478	0.316	-0.059
	Right vmPFC	-1.223	0.111	-0.179	**-2.349**	**0.010**	**-0.290**
Follow-up	Left vmPFC	**-2.292**	**0.012**	**-0.335**	**-3.392**	**4.0E-4**	**-0.418**
	Right vmPFC	**-1.869**	**0.032**	**-0.273**	**-1.815**	**0.035**	**-0.224**

Among Females

Summary Statistics

Clusters		Con Group (*n = 154*)		BL-S Group (*n = 97*)		FU-S Group (*n = 193*)	
		mean	*std*	*mean*	*std*	*mean*	*std*
Baseline	Left vmPFC	0.600	0.091	0.577	0.087	0.582	0.087
	Right vmPFC	0.485	0.072	0.476	0.073	0.475	0.082
Development	Left vmPFC	-4.84E-03	2.13E-03	-4.75E-03	2.04E-03	-4.95E-03	2.02E-03
	Right vmPFC	-3.00E-03	1.65E-03	-3.26E-03	1.61E-03	-3.92E-03	1.83E-03
Follow-up	Left vmPFC	0.584	0.082	0.552	0.091	0.564	0.081
	Right vmPFC	0.467	0.060	0.442	0.066	0.457	0.055

Group Comparison

Clusters		BL-S *vs* Con Group			FU-S *vs* Con Group		
		T	$p_{one\text{-}tailed}$	*Cohen's d*	*T*	$p_{one\text{-}tailed}$	*Cohen's d*
Baseline	Left vmPFC	**-1.701**	**0.045**	**-0.220**	**-2.034**	**0.021**	**-0.220**
	Right vmPFC	**-1.679**	**0.047**	**-0.218**	-1.081	0.140	-0.117
Development	Left vmPFC	0.398	0.345	0.052	-0.845	0.199	-0.091
	Right vmPFC	**-1.652**	**0.050**	**-0.214**	**-3.386**	**4.0E-4**	**-0.366**
Follow-up	Left vmPFC	**-1.792**	**0.037**	**-0.232**	**-1.974**	**0.025**	**-0.213**
	Right vmPFC	**-1.961**	**0.025**	**-0.254**	**-1.877**	**0.031**	**-0.203**

At the group-level analysis, the two-tailed p-values were provided. In the validation among the separate sex, the one tailed p-values were provided.

Table S2. Correlations between brain features and smoking

Brain Features	Lifetime occasions of smoking at Baseline among BL-S Group (*n* = *181*)		Lifetime occasions of smoking at follow-up among FU-S Group (*n* = *366*)	
	r	$p_{one\text{-}tailed}$	***r***	$p_{one\text{-}tailed}$
Left vmPFC volume at baseline	**-0.170**	**0.016**	**-0.109**	**0.020**
Right vmPFC volume at baseline	-0.070	0.188	-0.075	0.083
Left vmPFC Development	-0.018	0.411	-0.074	0.085
Right vmPFC Development	0.086	0.140	**-0.126**	**0.010**
**Among Males**	**BL-S Group** ***(n = 84)***		**FU-S Group** ***(n = 173)***	
Left vmPFC volume at baseline	**-0.194**	**0.035**	**-0.126**	**0.048**
Right vmPFC Development	0.118	0.157	**-0.124**	**0.050**
**Among Females**	**BL-S Group** ***(n = 97)***		**FU-S Group** ***(n = 193)***	
Left vmPFC volume at baseline	**-0.166**	**0.048**	**-0.102**	**0.070**
Right vmPFC Development	0.068	0.263	**-0.133**	**0.032**
**Conduct Problem Score, quantity of marijuana and drink in a row as additional regressors**				
Left vmPFC volume at baseline	**-0.157**	**0.025**	**-0.091**	**0.043**
Right vmPFC Development	0.076	0.184	**-0.090**	**0.044**
**Quantity of Smoking in Last 30 days at follow-up**	**BL-S Group (*n* = *181*)**		**FU-S Group (*n* = *366*)**	
Left vmPFC volume at baseline	**-0.159**	**0.019**	**-0.091**	**0.040**
Right vmPFC Development	0.019	0.403	**-0.093**	**0.026**
**Lifetime Occasions of Smoking more than 3 times**	**BL-S Group (*n* = *94*)**		**FU-S Group (*n* = *276*)**	
Left vmPFC volume at baseline	**-0.208**	**0.037**	**-0.107**	**0.040**
Right vmPFC Development	-0.107	0.183	**-0.135**	**0.014**

The one-tailed p-values were provided.

Table S3. The correlation between features and novelty/sensation seeking scores

Feature	Baseline occasions of smoking		Follow-up occasions of smoking		Baseline left vmPFC volume		Follow-up left vmPFC volume		Development of right vmPFC	
**Score**	***r***	***p***	***r***	***p***	***r***	***p***	***r***	***p***	***r***	***p***
**BL novelty seeking**	**0.197**	**2.6E-8**	**0.338**	**2.1E-22**	**-0.073**	**0.041**	-0.067	0.062	-0.005	0.896
**FU novelty seeking**	**0.148**	**3.3E-5**	**0.349**	**6.1E-24**	**-0.102**	**0.005**	**-0.152**	**1.0E-5**	-0.040	0.261
**BL sensation seeking**	**0.155**	**1.3E-5**	**0.199**	**1.8E-8**	-0.013	0.721	-0.019	0.600	-0.053	0.141
**FU sensation seeking**	**0.111**	**0.002**	**0.236**	**2.3E-11**	**-0.086**	**0.017**	**-0.126**	**2.2E-4**	**-0.127**	**2.0E-4**

The two-tailed p-values without multiple comparison adjustments were provided.

Table S4. The correlation between features and the components of novelty seeking from TCI

Feature	Baseline Sensation Seeking		Baseline occasions of smoking		Baseline left vmPFC volume	
**Component**	***r***	***FDRp***	***r***	***FDRp***	***r***	***FDRp***
Disorderliness vs. Regimentation	**0.062**	**0.081**	**0.150**	**8.6E-5**	**-0.141**	**9.6E-5**
Exploratory Excitability vs. Stoic Rigidity	**0.365**	**7.2E-26**	**0.136**	**1.5E-4**	-0.053	0.268
Impulsiveness vs. Reflection Total Score	**0.215**	**1.1E-9**	**0.100**	**0.005**	-0.049	0.224
Extravagance vs. Reserve Total Score	**0.287**	**2.9E-16**	**0.148**	**5.5E-5**	-0.024	0.505
Subitems of "Disorderliness vs. Regimentation" Component						
**tci044 'I like it when people can do whatever they want without strict rules and regulations.'**	**0.069**	**0.056**	**0.111**	**0.001**	**-0.079**	**0.025**
tci051 'I am usually able to get other people to believe me, even when I know that what I am saying is exaggerated or untrue.'	**0.175**	**7.5E-7**	**0.096**	**0.004**	0.057	0.105
tci077 'Even when most people feel it is not important, I often insist on things being done in a strict and orderly way.' (Reversely Coded)	**0.104**	**0.003**	-0.049	0.083	-0.022	0.532
**tci109 'I often break rules and regulations when I think I can get away with it.'**	**-0.019**	**0.596**	**0.236**	**1.6E-11**	**-0.074**	**0.038**
tci135 'I can usually do a good job of stretching the truth to tell a funnier story or to play a joke on someone.'	**0.266**	**3.2E-14**	-0.004	0.452	0.023	0.514
tci159 'I am not very good at talking my way out of trouble when I am caught doing something wrong.' (Reversely Coded)	**0.173**	**1.1E-6**	**0.077**	**0.015**	0.021	0.558
tci170 'I have some trouble telling a lie, even when it is meant to spare someone elses feelings.' (Reversely Coded)	0.011	0.763	0.052	0.070	0.020	0.576

At the component-level, the two-tailed p-values with the false discovery rate (FDR) adjustments were provided. At the subitem-level, the two-tailed p-values without multiple comparison adjustments were provided.

Table S5. The results of Mendelian randomisation analysis in IMAGEN cohort.

The correlation between the PRSs and the the corresponding phenotype.

Threshold	PRSs of Left vmPFC Volume				PRSs of Smoking				PRSs of Rule Breaking			
	BL Left vmPFC volume		FU Left vmPFC volume		BL Smoking		FU Smoking		BL Rule Breaking		FU Rule Breaking	
	r	$p_{one\text{-}tailed}$	*r*	$p_{one\text{-}tailed}$	*r*	$p_{one\text{-}tailed}$	*r*	$p_{one\text{-}tailed}$	*r*	$p_{one\text{-}tailed}$	*r*	$p_{one\text{-}tailed}$
0.05	**0.399**	**1.6E-29**	**0.147**	**7.7E-5**	0.208	9.5E-9	0.074	0.024	0.369	1.8E-24	0.182	9.5E-7
0.1	0.395	7.1E-29	0.149	6.4E-5	**0.225**	**5.7E-10**	**0.077**	**0.020**	0.373	5.1E-25	0.185	6.5E-7
0.2	0.392	2.4E-28	0.146	8.9E-5	0.229	2.7E-10	0.065	0.041	0.377	1.7E-25	0.188	4.1E-7
0.3	0.390	3.9E-28	0.145	9.3E-5	0.233	1.2E-10	0.065	0.041	0.381	4.8E-26	0.187	4.7E-7
0.4	0.390	4.1E-28	0.145	1.0E-4	0.235	9.0E-11	0.064	0.043	**0.384**	**1.9E-26**	**0.189**	**3.5E-7**
0.5	0.391	3.3E-28	0.146	9.1E-5	0.236	8.0E-11	0.068	0.034	0.383	2.3E-26	0.189	3.7E-7

The statistical results of Mendelian randomisation analysis.

Threshold	Left vmPFC PRS$_{(p < 0.05)}$ → Smoking		Smoking PRS$_{(p < 0.1)}$ → Left vmPFC		Left vmPFC PRS$_{(p < 0.05)}$ → Rule Breaking		Rule Breaking PRS$_{(p < 0.4)}$ → Left vmPFC		Rule Breaking PRS$_{(p < 0.4)}$ → Smoking		Smoking PRS$_{(p < 0.1)}$ → Rule Breaking	
	r	$p_{one\text{-}tailed}$	*r*	$p_{one\text{-}tailed}$	*r*	$p_{one\text{-}tailed}$	*r*	$p_{one\text{-}tailed}$	*r*	$p_{one\text{-}tailed}$	*r*	$p_{one\text{-}tailed}$
0.05	**-0.065**	**0.040**	-0.002	0.473	**-0.075**	**0.022**	-0.024	0.264	**0.068**	**0.033**	0.007	0.422
0.1	**-0.063**	**0.045**	-0.002	0.479	**-0.074**	**0.024**	-0.023	0.268	**0.067**	**0.036**	0.009	0.406
0.2	**-0.064**	**0.043**	-0.003	0.470	**-0.074**	**0.023**	-0.023	0.268	**0.067**	**0.036**	0.011	0.381
0.3	**-0.064**	**0.043**	-0.001	0.493	**-0.074**	**0.023**	-0.023	0.268	**0.067**	**0.036**	0.014	0.354
0.4	**-0.064**	**0.043**	0.002	0.484	**-0.074**	**0.023**	-0.024	0.259	**0.067**	**0.036**	0.016	0.335
0.5	**-0.061**	**0.050**	0.003	0.468	**-0.073**	**0.025**	-0.023	0.267	**0.066**	**0.039**	0.015	0.342

The one-tailed p-values without multiple comparison adjustments were provided.

Table S6. The results of Mendelian randomisation analysis on the other maladaptive behaviour.

The correlation between the PRSs and the the corresponding phenotype.

Threshold	PRSs of Conduct Problem				PRSs of Marijuana Used				PRSs of Drink in a Row			
	BL Conduct Problem		FU Conduct Problem		BL Marijuana Used		FU Marijuana Used		BL Drink in a Row		FU Drink in a Row	
	r	$p_{one\text{-}tailed}$	***r***	$p_{one\text{-}tailed}$	***r***	$p_{one\text{-}tailed}$	***r***	$p_{one\text{-}tailed}$	***r***	$p_{one\text{-}tailed}$	***r***	$p_{one\text{-}tailed}$
0.05	0.301	2.1E-16	0.076	0.043	0.122	0.002	0.079	0.041	0.204	5.8E-8	0.079	0.038
0.1	0.301	2.0E-16	0.074	0.049	0.138	3.4E-4	0.068	0.076	0.219	5.5E-9	0.092	0.015
0.2	0.307	4.5E-17	0.076	0.042	0.135	4.2E-4	0.072	0.063	0.224	2.4E-9	0.092	0.016
0.3	0.306	5.6E-17	0.077	0.040	**0.135**	**4.5E-4**	**0.081**	**0.036**	0.226	1.7E-9	0.099	0.009
0.4	0.306	5.6E-17	0.077	0.039	0.133	0.001	0.080	0.037	**0.228**	**1.2E-9**	**0.099**	**0.009**
0.5	**0.308**	**4.0E-17**	**0.078**	**0.037**	0.133	0.001	0.077	0.046	0.226	1.6E-9	0.096	0.011

The statistical results of Mendelian randomisation analysis.

Threshold	Left vmPFC PRS$_{(p < 0.05)}$ → Conduct Problem		Left vmPFC PRS$_{(p < 0.05)}$ → Marijuana Used		Left vmPFC PRS$_{(p < 0.05)}$ →Drink in a Row		Rule Breaking PRS$_{(p < 0.4)}$ → Conduct Problem		Rule Breaking PRS$_{(p < 0.4)}$ → Marijuana Used		Rule Breaking PRS$_{(p < 0.4)}$ →Drink in a Row	
	r	$p_{one\text{-}tailed}$	***r***	$p_{one\text{-}tailed}$	***r***	$p_{one\text{-}tailed}$	***r***	$p_{one\text{-}tailed}$	***r***	$p_{one\text{-}tailed}$	***r***	$p_{one\text{-}tailed}$
0.05	**-0.076**	**0.020**	**-0.068**	**0.038**	**-0.063**	**0.050**	**0.070**	**0.032**	**0.074**	**0.028**	**0.089**	**0.008**
0.1	**-0.076**	**0.021**	**-0.067**	**0.039**	**-0.063**	**0.049**	**0.069**	**0.032**	**0.073**	**0.029**	**0.090**	**0.008**
0.2	**-0.074**	**0.024**	**-0.067**	**0.040**	**-0.063**	**0.049**	**0.069**	**0.033**	**0.073**	**0.030**	**0.090**	**0.008**
0.3	**-0.072**	**0.026**	**-0.067**	**0.039**	-0.061	0.053	**0.067**	**0.037**	**0.071**	**0.033**	**0.090**	**0.008**
0.4	**-0.073**	**0.025**	**-0.066**	**0.041**	-0.061	0.054	**0.066**	**0.040**	**0.069**	**0.037**	**0.091**	**0.007**
0.5	**-0.073**	**0.024**	**-0.064**	**0.045**	-0.060	0.057	**0.065**	**0.041**	**0.068**	**0.038**	**0.090**	**0.008**

Threshold	Conduct Problem PRS$_{(p < 0.5)}$ → Left vmPFC		Conduct Problem PRS$_{(p < 0.5)}$ → Rule Breaking		Marijuana Used PRS$_{(p < 0.3)}$ → Left vmPFC		Marijuana Used PRS$_{(p < 0.3)}$ → Rule Breaking		Drink in a Row PRS$_{(p < 0.4)}$ → Left vmPFC		Drink in a Row PRS$_{(p < 0.4)}$ → Rule Breaking	
	r	$p_{one\text{-}tailed}$	***r***	$p_{one\text{-}tailed}$	***r***	$p_{one\text{-}tailed}$	***r***	$p_{one\text{-}tailed}$	***r***	$p_{one\text{-}tailed}$	***r***	$p_{one\text{-}tailed}$
0.05	-0.011	0.382	0.051	0.083	0.024	0.265	0.010	0.399	-0.019	0.305	0.033	0.196
0.1	-0.012	0.375	0.052	0.079	0.026	0.249	0.009	0.405	-0.020	0.296	0.029	0.222
0.2	-0.012	0.375	0.053	0.075	0.021	0.293	0.007	0.425	-0.019	0.305	0.026	0.244
0.3	-0.012	0.377	0.055	0.069	0.021	0.289	0.007	0.424	-0.018	0.315	0.024	0.265
0.4	-0.012	0.378	0.054	0.073	0.019	0.308	0.008	0.423	-0.021	0.290	0.027	0.243
0.5	-0.011	0.385	0.053	0.077	0.021	0.296	0.007	0.431	-0.022	0.282	0.025	0.259

The one-tailed p-values without multiple comparison adjustments were provided.

Table S7. The results of Mendelian randomisation analysis in ABCD cohort.

The correlation between the PRSs and the the corresponding phenotype.

Threshold	PRSs of Rule Breaking				PRSs of Left vmPFC	
	BL Rule Breaking		FU Rule Breaking		BL Left vmPFC volume	
	r	$p_{one\text{-}tailed}$	*r*	$p_{one\text{-}tailed}$	*r*	$p_{one\text{-}tailed}$
0.05	0.030	0.022	0.035	0.010	0.153	2.0E-24
0.1	0.034	0.012	0.033	0.014	0.138	4.0E-20
0.2	**0.028**	**0.030**	**0.038**	**0.006**	0.107	1.2E-12
0.3	0.023	0.060	0.034	0.012	0.136	1.4E-19
0.4	0.024	0.059	0.035	0.011	0.152	4.1E-24
0.5	0.023	0.067	0.038	0.006	**0.162**	**3.3E-27**

The statistical results of Mendelian randomisation analysis.

Threshold	Left vmPFC PRS$_{(p < 0.5)}$ → BL Rule Breaking		Left vmPFC PRS$_{(p < 0.5)}$ → FU Rule Breaking		Rule Breaking PRS$_{(p < 0.2)}$ →BL Left vmPFC volume	
	r	$p_{one\text{-}tailed}$	*r*	$p_{one\text{-}tailed}$	*r*	$p_{one\text{-}tailed}$
0.05	-0.024	0.056	**-0.029**	**0.024**	-0.012	0.223
0.1	**-0.025**	**0.049**	**-0.030**	**0.023**	0.003	0.413
0.2	**-0.027**	**0.039**	**-0.031**	**0.019**	0.001	0.485
0.3	**-0.027**	**0.037**	**-0.033**	**0.015**	0.002	0.456
0.4	**-0.025**	**0.048**	**-0.031**	**0.020**	0.002	0.442
0.5	**-0.025**	**0.048**	**-0.030**	**0.023**	0.002	0.447

The one-tailed p-values without multiple comparison adjustments were provided.

Table S8. Correlations with the PRS of left vmPFC in different datasets

Dataset	Age	Age Gap	Sample Size	r_{mean}	$p_{one\text{-}tailed}$
IMAGEN-BL*	14	0	737	0.393	5E-29
ABCD	9-10	~4.5	4390	0.141	3E-21
IMAGEN-FU	19	5	737	0.146	3E-5
IMAGEN-FU2	23	9	737	0.112	9E-4
HCP	22-37	~16	672	0.070	0.04
UKB1	40-50	~30	19346	0.011	0.06
UKB2	50-60	~40	16596	0.006	0.22
UKB3	> 60	~50	10145	0.004	0.34

*The same age as the discovery sample. The one-tailed p-values were provided.

Table S9. The Correlation between the development of right vmPFC and the longitudinal changes of subitems of sensation seeking scores

Subitems of sensation seeking score in the Substance Use Risk Profile Scale	All Sample (*n = 807*)		Con Group (*n = 260*)		BL-S Group (*n = 181*)		FU-S Group (*n = 366*)	
	r	*FDR* $p_{one\text{-}tailed}$	*r*	$p_{one\text{-}tailed}$	*r*	$p_{one\text{-}tailed}$	*r*	$p_{one\text{-}tailed}$
surps3 'I would like to skydive.'	-0.019	0.375	0.039	0.275	-0.035	0.329	-0.024	0.327
**surps6 'I enjoy new and exciting experiences even if they are unconventional.'**	**-0.112**	**0.004**	0.044	0.248	**-0.144**	**0.036**	**-0.221**	**2.0E-5**
**surps9 'I like doing things that frighten me a little.'**	**-0.097**	**0.008**	0.066	0.156	-0.079	0.163	-0.063	0.122
surps12 'I would like to learn how to drive a motorcycle.'	-0.005	0.443	0.000	0.499	0.048	0.276	-0.023	0.339
surps19 'I would enjoy hiking long distances in wild and uninhabited territory.'	-0.042	0.200	0.021	0.371	-0.035	0.330	-0.042	0.218

For the correlations obtained from all samples, the one-tailed p-values with the false discovery rate (FDR) adjustments were provided. In each group, the one-tailed p-values without multiple comparison adjustments were provided.

Table S10. The correlation between brain features and smoking dependence scores among the regular smokers from the HCP dataset.

Tobacco Use and Dependence Score	L-vmPFC		R-vmPFC		Difference	
	r	$p_{one\text{-}tailed}$	*r*	$p_{one\text{-}tailed}$	*Steiger's Z*	*p*
FTND Total Score	-0.057	0.173	**-0.135**	**0.012**	**2.243**	**0.025**
Age first smoked a cigarette (even a puff)	**0.153**	**0.005**	0.084	0.061	**1.989**	**0.047**
DSM tobacco dependence - withdrawal	-0.008	0.448	**-0.119**	**0.025**	**3.184**	**0.001**

For the association analysis, the one-tailed p-values without the multiple comparison adjustments were provided. The differences of correlations were evaluated with Steiger’s Z tests and the two-tailed p-values were provided.
