# Supplementary material for: Association between vmPFC gray matter volume and smoking initiation in adolescents
Source: Nat Commun. 2023 Aug 15;14:4684. doi: 10.1038/s41467-023-40079-2 (PMC10427673; doi:10.1038/s41467-023-40079-2)
Supplement: Supplementary file 3 — Reporting Summary [file 41467_2023_40079_MOESM3_ESM.pdf]

nature portfolio

Corresponding author(s): Tianye Jia; Jianfeng Feng; Trevor W. Robbins.

Last updated by author(s): May 21, 2023

Reporting Summary

Nature Portfolio wishes to improve the reproducibility of the work that we publish. This form provides structure for consistency and transparency in reporting. For further information on Nature Portfolio policies, see our Editorial Policies and the Editorial Policy Checklist.

Statistics

For all statistical analyses, confirm that the following items are present in the figure legend, table legend, main text, or Methods section.

n/a	Confirmed	
☐	☒	The exact sample size (*n*) for each experimental group/condition, given as a discrete number and unit of measurement
☐	☒	A statement on whether measurements were taken from distinct samples or whether the same sample was measured repeatedly
☐	☒	The statistical test(s) used AND whether they are one- or two-sided<br>*Only common tests should be described solely by name; describe more complex techniques in the Methods section.*
☐	☒	A description of all covariates tested
☐	☒	A description of any assumptions or corrections, such as tests of normality and adjustment for multiple comparisons
☐	☒	A full description of the statistical parameters including central tendency (e.g. means) or other basic estimates (e.g. regression coefficient) AND variation (e.g. standard deviation) or associated estimates of uncertainty (e.g. confidence intervals)
☐	☒	For null hypothesis testing, the test statistic (e.g. *F*, *t*, *r*) with confidence intervals, effect sizes, degrees of freedom and *P* value noted<br>*Give P values as exact values whenever suitable.*
☒	☐	For Bayesian analysis, information on the choice of priors and Markov chain Monte Carlo settings
☒	☐	For hierarchical and complex designs, identification of the appropriate level for tests and full reporting of outcomes
☐	☒	Estimates of effect sizes (e.g. Cohen's *d*, Pearson's *r*), indicating how they were calculated

Our web collection on statistics for biologists contains articles on many of the points above.

Software and code

Policy information about availability of computer code

Data collection	Data collections of IMAGEN project, ABCD and HCP datasets were done centrally, not performed by us.
Data analysis	Open source software was used for this study including Matlab R2020b, SPM12, R v3.5.1 and METAL software. The details of data preprocessing and analysis as well as the software used were described in the Methods. Custom code that supports the findings of this study is available here (https://github.com/Shitong-Xiang/Smoking.git). Additional information related to this paper may be requested from the authors.

For manuscripts utilizing custom algorithms or software that are central to the research but not yet described in published literature, software must be made available to editors and reviewers. We strongly encourage code deposition in a community repository (e.g. GitHub). See the Nature Portfolio guidelines for submitting code & software for further information.

Data

Policy information about availability of data

All manuscripts must include a data availability statement. This statement should provide the following information, where applicable:

- Accession codes, unique identifiers, or web links for publicly available datasets
- A description of any restrictions on data availability
- For clinical datasets or third party data, please ensure that the statement adheres to our policy

The IMAGEN project data are available from a dedicated database: https://imagen-project.org.

ABCD data are available from a dedicated database: https://abcdstudy.org.
HCP data are available from a dedicated database: https://www.humanconnectome.org.

Data availability:
The IMAGEN project data are available from a dedicated database: https://imagen-project.org. ABCD data are available from a dedicated database: https://abcdstudy.org. HCP data are available from a dedicated database: https://www.humanconnectome.org. All data needed to evaluate the conclusions in this study are present in the paper and/or the Supplementary Informations. Source data are provided with this paper.

Human research participants

Policy information about studies involving human research participants and Sex and Gender in Research.

Reporting on sex and gender	The term sex (biological attribute, i.e., female or male) was used in this study. In the discover dataset (i.e., IMAGEN project), there was no significant difference in sex between smokers (both BL-S and FU-S) and controls. Sex was regressed out as a covariate in all subsequent analyses.
Population characteristics	The demographic characteristics of these participants are summarized in Table 1. Sex, handedness, IQ, research sites, body mass index (BMI), total intracranial volume (TIV), socioeconomic score and negative life events were regressed out as covariates.
Recruitment	Data collections of IMAGEN project , ABCD and HPC datasets were done centrally, not performed by us.
Ethics oversight	The IMAGEN study has been initially funded by the European Commission, and has subsequently received funding from various agencies including the European Research Council (ERC), Medical Research Council (MRC), National Institute for Health Research UK (NIHR), Swedish Research Council (Vetenskapsrådet), German Federal Ministry of Research & Education (BMBF), National Institute for Health Research US (NIHR) and the National Institute on Drug Abuse (NIDA). ABCD and HCP dataset was supported by the National Institutes of Health (NIH). Informed consent was sought from all participants and/or a parent/guardian of each participant.

Note that full information on the approval of the study protocol must also be provided in the manuscript.

Field-specific reporting

Please select the one below that is the best fit for your research. If you are not sure, read the appropriate sections before making your selection.

☒ Life sciences ☐ Behavioural & social sciences ☐ Ecological, evolutionary & environmental sciences

For a reference copy of the document with all sections, see nature.com/documents/nr-reporting-summary-flat.pdf

Life sciences study design

All studies must disclose on these points even when the disclosure is negative.

Sample size	Total 807 participants with complete longitudinal structural images and behavioural scores from IMAGEN project were included in the initial analysis. For genetic analysis, the remaining 1026 subjects from the IMAGEN project were also included and 4417 participants from ABCD dataset were included to validate the relationship between the GMV of left vmPFC and rule-breaking behaviour and discover the genetic factors underlying the neurobehavioural circuit.
Data exclusions	For IMAGEN project, participants lacking longitudinal follow-up data and/or complete behavioural data were excluded first. The neuroimage data with poor registration and segmentation (by visual check) were also excluded. For ABCD dataset, only self-reported white people with the quality-controlled brain images at baseline year and complete behavioural data at both baseline year and one-year follow-up interview were included to ensure homogeneity of the datasets. For HCP dataset, only regular/daily smokers with the quality-controlled brain images and complete behavioural data were invulded as an additional validation.
Replication	No replication was conducted given the huge sample size of the present study
Randomization	As a population study, no randomization was conducted. However, covariates were regressed out in the analyses.
Blinding	As a population-based observational study, the IMAGEN project does not focus on any specific exposures of outcomes and no intervention or treatment was applied. Therefore, blinding was not necessary for sampling.

Reporting for specific materials, systems and methods

We require information from authors about some types of materials, experimental systems and methods used in many studies. Here, indicate whether each material, system or method listed is relevant to your study. If you are not sure if a list item applies to your research, read the appropriate section before selecting a response.

Materials & experimental systems

n/a	Involved in the study
☒	☐ Antibodies
☒	☐ Eukaryotic cell lines
☒	☐ Palaeontology and archaeology
☒	☐ Animals and other organisms
☒	☐ Clinical data
☒	☐ Dual use research of concern

Methods

n/a	Involved in the study
☒	☐ ChIP-seq
☒	☐ Flow cytometry
☐	☒ MRI-based neuroimaging

Magnetic resonance imaging

Experimental design

Design type: N/A

Design specifications: N/A

Behavioral performance measures: N/A

Acquisition

Imaging type(s): Structural MRI

Field strength: 3 Tesla

Sequence & imaging parameters: High-resolution 3-dimensional T1-weighted images were acquired with a gradient-echo sequence. The details of sequence parameters were provided in Methods.

Area of acquisition: Whole brain scan

Diffusion MRI: ☐ Used ☒ Not used

Preprocessing

Preprocessing software: Processing of the structural T1-weighted images was performed with the Statistical Parametric Mapping toolbox (SPM, Wellcome Department of Neuroimaging, London, United Kingdom; http://www.fil.ion.ucl.ac.uk/spm). For voxel-based morphometry (VBM), the images were first segmented into gray matter, white matter and cerebrospinal fluid tissue maps with the VBM8 toolbox (http://www.neuro.uni-jena.de/vbm). Then the gray matter maps were used to generate the study-specific template by the DARTEL algebra. Next, the gray matter maps were warped to standard Montreal Neurological Institute (MNI) space (1.5*1.5*1.5 mm resolution) with an iterative registration and modulated by multiplying the linear and nonlinear components of the Jacobian determinants. Finally, the gray matter maps were smoothed with a full width at the FWHM Gaussian kernel of 8 mm, and the value of each voxel was absolute gray matter volume (GMV). For gray matter development, both the BL and FU scans from each participant were first processed with the pairwise longitudinal tool in SPM to generate a within-subject average template and the corresponding Jacobian determinants maps. Then the within-subject average templates were segmented and warped to the standard MNI space with the same pipeline as VBM. Next, the native space gray matter maps from the within-subject average template were multiplied voxel-by-voxel with the corresponding Jacobian determinants maps to obtain the gray matter volumetric changes rate maps. The transform fields were then applied to the gray matter volumetric change rate maps to normalise it to standard MNI space. Finally, the gray matter volumetric changes rate maps were also smoothed with a full width at an FWHM Gaussian kernel of 8 mm. The value of each voxel represented the voxel-wise volumetric changes rate per year.

Normalization: Nonlinearly normalized to MNI space.

Normalization template: MNI space

Noise and artifact removal: N/A

Volume censoring: N/A

Statistical modeling & inference

Model type and settings: N/A

Effect(s) tested: The linear regression and T-tset was applied.

Specify type of analysis: ☒ Whole brain ☐ ROI-based ☐ Both

Statistic type for inference (See Eklund et al. 2016): Voxel-wise comparisons were performed and FWE correction was applied for cluster-wise correction.

Correction | FWE correction was applied to identify the significant clusters. Permutation correction was applied wherever applicable.

Models & analysis

n/a	Involved in the study
☒	☐ Functional and/or effective connectivity
☒	☐ Graph analysis
☒	☐ Multivariate modeling or predictive analysis
